# Notes and Personals

**Published:** 1898-07

**Authors:** 


### CANADIAN NOTES AND PERSONALS.

Toronto, on May 7th, was the scene of a splendid horse-show and military tournament.

Influenza prevailed in epizöotic form throughout the province of Manitoba during the spring months to quite a marked extent.

Good drivers and saddlers are in great demand in Ottawa, and are bringing good prices.

The city of Ottawa adopted an ordinance last fall making it compulsory for the dairies supplying milk to the city to furnish a certificate from a qualified veterinarian that their herds had been tested with tuberculin and that all were in healthy condition. Much credit is due to Drs. James and Perley, of Ottawa, Government veterinary inspectors, for the accomplishment of this very much to be desired movement.

Dr. D. K. Smith, of Toronto, is now in London, England, attending the leading hospitals and taking a special course in pathology under Prof. McFaydean.

Dr. Fred. Torrance, of Winnipeg, has been selected as one of the judges of horses at the Provincial Exhibition of British Columbia, to be held next October at New Westminster.

Dr. A. B. Campbell, of Berlin, reports a very busy season of practice.

Dr. J. A. Graham, of Claremont, will judge the Clydes, Shires, draughts, and general-purpose horses; Dr. Quinn, of Brampton, will judge standard breds, coachers, and roadsters, at the Winnipeg Industrial Exposition.

Dr. R. R. Dinwiddie, class of 1886 of the Ontario Veterinary College, has just issued from the Arkansas Experiment Station a bulletin on "Methods of Combating Communicable Diseases of Farm-animals."

Dr. Fred. Torrance, of Winnipeg, spent a week in June addressing Farmers' Institutes of the Province of Manitoba.

Dr. James A. Waugh, of Pittsburg, Pa., graduate of Ontario Veterinary College, is the happy parent of a new boy baby who has already been named for Dr. William J. Waugh, of the U. S. Army.

Veterinary Surgeon John Robertson, graduate of the Veterinary Department of the McGill University, holding the rank of second lieutenant of the Sixth U. S. Infantry, is on the official list of wounded sent by General Shafter to Washington ; he was formerly a member of Troop C, Second Cavalry, until appointed by the President on April 12th for commission. Dr. Robertson is well known to many members of the profession, having for years filled the position of an army veterinarian, but, tiring of the prospects of receiving a commission as a veterinarian, resigned his place and enlisted as a private and was advanced to the position of troop sergeant, and later to that of second lieutenant.

Dr. C. E. Elliott, of St. Catharine's, was the judge of the standard-bred stallions at the horse-show in Toronto in May.

Dr. W. A. Dunbar, of Winnipeg, Manitoba, answers many of the cases reported for advice in the *Farmers' Advocate*, of London, Ont.

Dr. A. G. Hopkins, of Neepawa, Manitoba, was a visitor to the East in the spring, having placed Dr. Will A. Hillary in charge of his practice.

Dr. Wilson Swenerton, formerly of Wawanesa, has removed to Carberry, Manitoba.

Dr. J. G. Rutherford, of the House of Commons of Ottawa, Canada, a representative veterinarian of Manitoba, is a hard worker and faithful advocate of the people's interests in provincial government.

Veterinarian C. A. Sankey, of Lowville, N. Y., a graduate of the Ontario Veterinary College, contributed an interesting article on "The Housing of Livestock, with Regard to Health," to the June 15th issue of the *Farmers' Advocate*, of London, Ontario.

Dr. C. L. Morin, of St. Albans, Vt., was a visitor to Ottawa during the latter part of June.

Dr. A. E. James, Government Veterinary Inspector, of Ottawa, accompanied the 43d Battallion to Burlington, Vt., on July 4th.

Dr. H. S. Perley, late inspector for the Vermont Cattle Commission, now of Ottawa, Ontario, has just returned from a trip to Vermont and New Hampshire.

